# How Shall I Count the Ways? A Method for Quantifying the Qualitative Aspects of Unscripted Movement With Laban Movement Analysis

**DOI:** 10.3389/fpsyg.2019.00572

**Published:** 2019-03-28

**Authors:** Rachelle Palnick Tsachor, Tal Shafir

**Affiliations:** ^1^School of Theatre and Music, The University of Illinois at Chicago, Chicago, IL, United States; ^2^The Emili Sagol Creative Arts Therapies Research Center, University of Haifa, Haifa, Israel; ^3^Department of Psychiatry, University of Michigan, Ann Arbor, MI, United States

**Keywords:** Laban Movement Analysis, movement, bodily emotional expressions, dance/movement therapy, motion analysis, Laban/Bartenieff Movement System, movement quality, non-verbal behavior

## Abstract

There is significant clinical evidence showing that creative and expressive movement processes involved in dance/movement therapy (DMT) enhance psycho-social well-being. Yet, because movement is a complex phenomenon, statistically validating which aspects of movement change during interventions or lead to significant positive therapeutic outcomes is challenging because movement has multiple, overlapping variables appearing in unique patterns in different individuals and situations. One factor contributing to the therapeutic effects of DMT is movement’s effect on clients’ emotional states. Our previous study identified sets of movement variables which, when executed, enhanced specific emotions. In this paper, we describe how we selected movement variables for statistical analysis in that study, using a multi-stage methodology to identify, reduce, code, and quantify the multitude of variables present in unscripted movement. We suggest a set of procedures for using Laban Movement Analysis (LMA)-described movement variables as research data. Our study used LMA, an internationally accepted comprehensive system for movement analysis, and a primary DMT clinical assessment tool for describing movement. We began with Davis’s (1970) three-stepped protocol for analyzing movement patterns and identifying the most important variables: (1) We repeatedly observed video samples of validated (Atkinson et al., 2004) emotional expressions to identify prevalent movement variables, eliminating variables appearing minimally or absent. (2) We use the criteria repetition, frequency, duration and emphasis to eliminate additional variables. (3) For each emotion, we analyzed motor expression variations to discover how variables cluster: first, by observing ten movement samples of each emotion to identify variables common to all samples; second, by qualitative analysis of the two best-recognized samples to determine if phrasing, duration or relationship among variables was significant. We added three new steps to this protocol: (4) we created Motifs (LMA symbols) combining movement variables extracted in steps 1–3; (5) we asked participants in the pilot study to move these combinations and quantify their emotional experience. Based on the results of the pilot study, we eliminated more variables; (6) we quantified the remaining variables’ prevalence in each Motif for statistical analysis that examined which variables enhanced each emotion. We posit that our method successfully quantified unscripted movement data for statistical analysis.

## Introduction

Body movement is becoming ever more recognized as integral to both mental and physical health (Penedo and Dahn, 2005; Bradt et al., 2015; Liao et al., 2015; Teychenne et al., 2017). Dance/movement therapy (DMT) uses body movement as a primary tool to support and improve psycho-social well-being through creative and expressive movement processes. DMT is a well-established therapy with clinical evidence of effectiveness (Ritter and Low, 1996; Strassel et al., 2011; Kiepe et al., 2012; Koch et al., 2014; Lee et al., 2015; Sossin, 2018). One of the factors contributing to the therapeutic effects of DMT is the effect of movement on the client’s emotional state. Researching which aspects of movement are responsible for the effects of different movements on specific emotions is challenging, because unscripted movement in individuals or groups is difficult to quantify for statistical analysis. Actual observable human movement is a complex phenomenon, with multiple, overlapping variables, uniquely presenting in each individual. Only a sophisticated observation and descriptive method can do justice to movement’s content and context (Sossin, 2018). And, even with such an observation tool, the number of variables in movement is so large that the research design for each study needs to include a way to reduce the number of variables being analyzed to a smaller set of variables that can be tested statistically, as well as to quantify them in ways that capture the essential expression and context.

The problem is that of methodology: much of the research on the relationship between movement and emotion has been on static posture and positions, rather than on the movements in between those postures and positions (e.g., Cuddy et al., 2018). Movement has been difficult to investigate quantitatively due to the enormous number of variables involved. Complete description of all movement variables is cumbersome and some selection is necessary to examine a given problem (Davis, 1970). Thus, the first challenge movement researchers face is to identify and extract the variables most significant for that study. This paper outlines the methodology we used for identifying, coding and quantifying the multitude of variables present in ordinary (unscripted) movement, so statistical analysis could be used to identify which specific variables are significant for emotion elicitation. The same or similar methodology might be used in the future for other studies involving complex natural unscripted movements, such as studies examining motor emotional expressions, or movements that characterize specific populations (e.g., children with developmental coordination disorders), etc. Therefore, the purpose of this paper is to suggest a set of procedures for preparing Laban Movement Analysis (LMA)-described movement data for statistical analysis.

### Existing Methods for Analyzing Complex Movement

Researchers in numerous fields have developed a variety of methods to analyze movement behavior, many of which were devised to suit the purpose of that particular research. The use of so many systems has made it difficult to compare results and improve methods (Davis, 1975; Levy and Duke, 2003; Shafir et al., 2016). Additionally, many of these systems have limitations: Some researchers, such as Darwin (1872), Wallbott (1998), or Dael et al. (2012) identified several specific movements executed with specific body parts and examined the existence of those movements within natural movements. These systems are limited to the defined movements they can study. Other researchers used coding systems of various movement dimensions such as: vertical and sagittal directions, force, velocity, and directness (de Meijer, 1989); direction, force, tempo and form (Montepare et al., 1999) or they characterized movements by the specific muscles that are activated (Huis in ’t Veld et al., 2014a,b) or kinematic variables such as joint displacement, velocity, and acceleration, or joint coordination (Pollick et al., 2001; Sawada et al., 2003; Roether et al., 2009; Gross et al., 2010, 2012; Barliya et al., 2013). These systems often cannot sufficiently track changes in qualitative aspects of unscripted movement, limiting their use for studying movement in contexts different from their study. The problem that was succinctly expressed by Levy and Duke (2003) still exists: that many movement analysis systems are designed to gather easily quantified empirical evidence, skewing research to what is easily studied, rather than what might be significant, yet difficult to quantify for scientific analysis.

### Laban Movement Analysis as a Research Instrument

Our method is based in LMA, an internationally accepted comprehensive system naming movement components. LMA, and systems emerging from it, have a strong record of inter-observer reliability when applied carefully by raters trained for the research project (Davis, 1981; Winter, 1992; Levy and Duke, 2003; Connors and Rende, 2018; Melzer et al., 2018). It has long been used to study emotional expression and as a primary clinical assessment tool in DMT (Davis, 1981; Cruz, 2009; Goodill et al., 2017). LMA is often used in research for its ability to capture qualitative aspects of movement as components shift over time or remain constant, and for identifying patterns. It affords users the capacity to recognize movement themes at both the macro and micro-analysis levels, and is valued for its accuracy in observing subtle and momentary motor changes, for its comprehensiveness in noting both functional and expressive aspects of movement, and for its language-based descriptors, which align with how people think about movement (e.g., ‘touch lightly’ as compared to measurements generated from a pressure sensor). While many have contributed to the development of LMA as an observational tool, and contemporary scholars often use the more current term “Laban/Bartenieff Movement System” (LBMS) to reflect the contributions of Irmgard Bartenieff to the comprehensiveness of the system, we chose to stay with the label LMA, so researchers from disparate fields (such as animation, human-machine interface, affective computing, and machine learning) can compare aspects from all Laban-based systems.

### The LMA System

Laban Movement Analysis is adept at describing what moves, where it moves, how it moves, and the ‘why’ of movement, in the relationship of the mover to self, others and the environment (Davis, 1970). The smallest (irreducible) movement units described by LMA are components of four main movement categories: Body, Space, Shape, and Effort (Davis, 1970; Bartenieff and Lewis, 1980). The Body category describes “what is moving,” e.g., which body parts are moving, and the coordination of these parts as well as basic actions such as walking or jumping. The Space category describes “where the body moves,” such as the direction of a movement (up/down, forward/backward, sideways open or across), planes the movement occurs in (vertical, sagittal, and horizontal), as well as use of the Kinesphere (e.g., far-reach space, peripheral movement), and more. The Shape category can describe changes in the shape of the body itself, moving in relation to one’s surroundings, to others and to one’s own needs, often reflecting the “why” of movement. We observe Shape when we note such things as whether a body encloses or spreads, rises or sinks. The last movement category is Effort, describing “how the body moves”: the qualitative aspects of movement such as lightly, suddenly, freely, etc. Effort reflects the mover’s inner attitude toward the movement as manifested in four different Factors: Weight, Space, Time, and Flow, each spanning two opposite polarities. Weight-Effort spans between the poles of *Strong* and *Light* and refers to the amount of force invested in the movement. Space-Effort ranges between *Direct* and *Indirect* and refers to the attitude toward the movement’s direction or focus. Time-Effort spans from *Sudden* to *Sustained*, reflecting the acceleration and deceleration of movement. Flow-Effort expresses the mover’s attitude toward controlling the progression of movement, from a higher control–*Binding* to little control or moving with abandon–*Freeing* (Studd and Cox, 2013).

### Advantages of the LMA Symbolic System for Identifying and Isolating Variables

**Figure 1:**
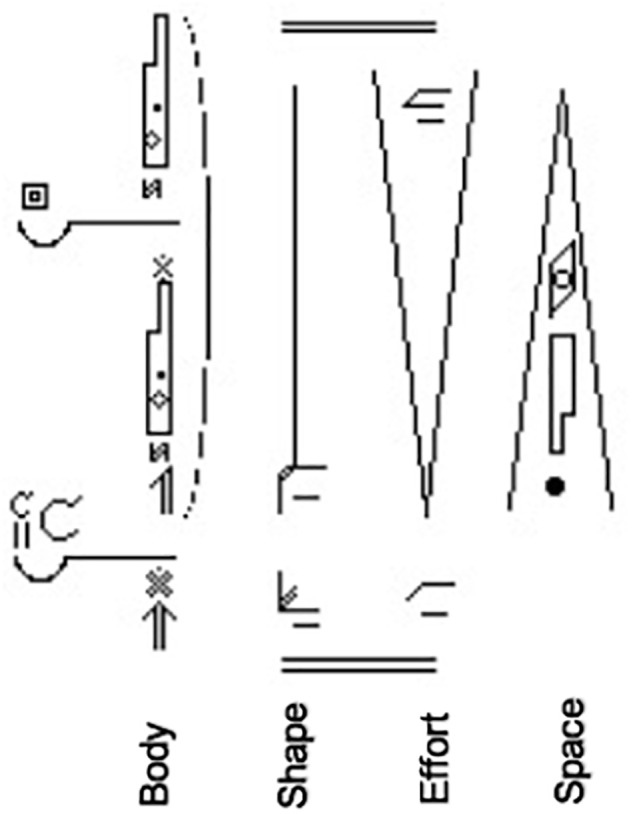
This Motif of an Atkinson video-clip depicts Body, Shape, Effort, and Space components in separate columns. Readers can move these components in their own way. Motif is read from the bottom up, with the lower double line marking the beginning of the movement. The first symbols above the double line are short, thus are brief in duration, and show the preparation part of the movement phrase. The duration of the symbols from the middle to the top/end of the Motif is extended in length (and therefore in time) by strokes and bows attached to them, showing the main action part of the phrase. The double bar at the top marks the end of movement.

One advantage of LMA is its unique system for writing movement through symbols called Motif Writing, which has been used to annotate behavior in both animal (Foroud and Pellis, 2003; Whishaw et al., 2003; Alaverdashvili et al., 2008) and human studies (Foroud and Whishaw, 2006, 2012). A Motif can flexibly capture not only movement components (written horizontally, like music notation), but also nuanced ways in which they appear in movement (written vertically to show duration and overlaps in timing), so non-component variables such as the phrasing of movement can be noted. A Motif provides symbolic representation of the movement which can be read directly, similar to reading music from notes, or math from numbers (Figure 1). Using Motif eliminates the need to show to study participants the movement to be performed. The advantage is that instruction for the execution of a movement sequence (a) generates clean data, and (b) allows us to encode and compare data from different study participants, each moving their own individual unique and slightly different movements, even though the movements by all participants include the same movement components (e.g., the component Light-Effort might be expressed in a hand gesture, a movement of the head, or lift of the chest).

(a) Clean data: when moving directly from Motif, participants generate movements that are uncontaminated by co-occurring movement components that might be unintentionally introduced through live or recorded motor demonstration. Similar to a musical score, which describes how to play sequences and combinations of notes over time, and may include both the notes to be played and the dynamics or expressive quality intended by the composer, Motif may include the Body parts doing the movement and their actions, the change in the mover’s Shape, the qualitative dynamics of Effort, and the advancement of the movement through Space.

(b) Motif allows us to encode and compare data from unscripted, individually unique movements of different people. When participants read and move Motifs of single components, combinations and sequences of components, they can move them in their own way, as long as the movement includes the required components. This enables researchers to isolate and analyze basic qualitative aspects of movement in any movement having the same components.

Laban Movement Analysis is increasingly used for movement research across disciplines precisely because of its ability to describe both quantitative and qualitative aspects of any movement. From its earliest development as a descriptive language, LMA has been used as a research tool for: assessing and treating polio (Bartenieff, 1955), rehabilitation (Bartenieff, 1962), cross-cultural studies (Bartenieff and Paulay, 1970), personality assessment (Eisenberg et al., 1972), for efficiency at work (Laban and Lawrence, 1974), performance style analysis (Bartenieff et al., 1984), and assessing psychiatric client behavior (Davis, 1981). The versatility of LMA (and systems emerging from it) is evident from its use in diverse types of research: it has been used to evaluate fighting behaviors of rats (Foroud and Pellis, 2003), to analyze behavior of non-human animals in naturalistic settings (Fagen et al., 1997), to diagnose autistic individuals (Dott, 1995), to evaluate motor recovery of stroke patients (Foroud and Whishaw, 2006), and to characterize the development of infants’ reaching movements (Foroud and Whishaw, 2012). Several studies have also used LMA-based systems to describe, recognize or create bodily emotional expressions for applications in human-robot interactions, interactive games such as the Xbox, and in animations (Camurri et al., 2003; Zhao and Badler, 2005; Rett et al., 2009; Barakova and Lourens, 2010; Zacharatos et al., 2013); to identify the brain mechanisms underlying the production of some of the LMA motor elements (Cruz-Garza et al., 2014), to compare expression in musicians (Broughton and Davidson, 2014, 2016), to study emotion recognition (Melzer et al., 2018) and to capture individual differences in decision-making style (Connors et al., 2014, 2016; Connors and Rende, 2018).

Yet, even with LMA and systems emerging from it, such as the Kestenberg Movement Profile (KMP), Action Profiling (AP), or Movement Pattern Analysis (MPA), quantifying data from non-prescribed movement demands expert observers and multiple steps of investigation (Connors et al., 2014; Connors and Rende, 2018; Sossin, 2018; Melzer et al., 2018). Researchers who have approached this challenge have slowly built a record of methods solving their unique research goals, and established the validity of using the system to encode data in computational movement studies, behavioral sciences, animal studies and cultural studies, as detailed in the next section.

## Quantifying Movement Variables

In computational modeling of movement, such as in robotics, motion capture and animation, a review of computable descriptive expressors of human motion found LMA to be the most comprehensive analysis system (Larboulette and Gibet, 2015). While many in these and similar fields (e.g., animal behavior) continue to improve quantification of movement data using LMA, there are still gaps. The first gap is found in papers using LMA theory without describing how they extract the LMA features, so their methods can’t be replicated (e.g., Woo et al., 2000; Foroud and Pellis, 2003; Whishaw et al., 2003; Alaverdashvili et al., 2008). Others didn’t actually observe movement, relying instead upon existing research in psychology and non-verbal communication to provide generalities about movements to set LMA-related parameters (e.g., Badler et al., 2002). Some relied upon LMA experts to create a fixed repertoire of motions as a training set for their systems, but their systems were untested on general movement, or relied upon comparing varying expressions of the same repeated movements (Bouchard and Badler, 2007; Castellano, 2008). In the last decade, much progress has been made toward automated recognition of LMA components (Rett and Dias, 2007; Rett et al., 2009; Santos and Dias, 2010; Bernstein et al., 2015a,b), but these are still not as capable as expert observers in capturing data from both unscripted movements and multiple movers, as a sampling of 2018 publications shows: Inthiam et al. (2018) recorded upper-body movements with a Kinect, to analyze for LMA features expressing emotion (taken from Shafir et al., 2016) in order to elicit critical parameters for generating robotic movement. However, their automatic assessment is limited to speed and Shape, and a human observer evaluated the consistency with the target emotion. Souza and Freire (2018) used Kinect to attempt to identify LMA Basic Effort Actions, testing only for 3 Effort factors in only one mover—and their accuracy isn’t described. Dewan et al. (2018) built a motion descriptor for emotion expression based on LMA which achieved 87% recognition. However, this was achieved by using a database of previously annotated skeletal *poses* of 3-d body-joints of only 6 gestural Body parts, and not by using *movement*. While their system identified Space, Shape and some Effort components, in order to achieve this result, they note that the dataset was annotated by “3 groups of observers,” for which no details of methods of observation are given. Bernhardt and Robinson’s (2007) computational model for emotion detection relies upon a data set of fixed actions: knocking, throwing, lifting and walking motion. They note that their system was impeded by the fact that “different people tend to display the same emotion in very different ways, so its ability to distinguish between sad and happy was smaller than hoped for.”

In the behavioral sciences, sophisticated, systematic methods for expert observers using LMA to code complex, unscripted movement behavior in individuals were laid out as early as the 1970s (Davis, 1970, 1981; Davis and Hadiks, 1994). More recently, Cruz and Koch (2004) wrote guidelines for movement observation using LMA (and systems emerging from it) in DMT research and clinical practice, noting how Davis (1987) already laid out the intricacies necessary to define the coding system. However, these methods don’t fully solve the quantification problem for statistical analysis of multiple movers and most researchers still use LMA-based systems for case studies or gather data from only some LMA aspects, such as Effort or Shape (Gross et al., 2010; Crane and Gross, 2013; Perugia et al., 2018).

Quantification of LMA components has often been attempted with various degrees of success for Body, Space, and Shape, but has been elusive without human observers. This is especially true for the Effort category, which describes qualitative aspects of movement. Development of a system capable of quantifying the majority of LMA components in unscripted (natural or improvised) movements of multiple people has remained a challenge, although researchers have made progress on quantification of limited sets of components (Davis, 1981; Gross et al., 2010, 2012; Samadani et al., 2013; Aristidou et al., 2015; Bernstein et al., 2015a,b) or in situations of prescribed movements (Nakata et al., 2002; Hachimura et al., 2005; Torresani et al., 2007; Truong and Zaharia, 2017) or used averaging to overcome the noisy data, and were unable to quantify data for statistical analysis (e.g., Senecal et al., 2016).

## Our Methodology

We devised our method for the study *Emotion Regulation through Movement* (Shafir et al., 2016) in order to discover which movement components contribute to the enhancement and/or evocation in people of each of four emotions: happiness, sadness, fear, and anger. Atkinson et al. (2004) had already found that basic emotions are readily identifiable from body movements, and that exaggeration of body movement enhanced recognition accuracy, producing higher emotional-intensity ratings indicating, that emotional intensity judgments of expressive body gestures rely more on movement than static form information. Shafir et al. (2013) went further, demonstrating that when participants (who did not know these movements were emotion-related) performed the specific movements from Atkinson’s clips, they experienced the same emotion as portrayed.

Yet, while numerous studies like these confirmed that emotions can be recognized from movement, and that movement affects emotions (e.g., Gross et al., 2010, 2012), identifying predominant movement parameters which were recognized or elicited emotion in unscripted movement remained a challenge. Atkinson’s study generated a set of validated clips that were ideal for this next challenge, so we began our study with a subset of his video clips. In these clips, 10 masked actors (5 male, 5 female) portrayed each of 4 basic emotions, Happiness, Sadness, Fear, and Anger, making a total of 40 clips. As in life, each actor expressed each target emotion in an individually unique way, so each of the 10 clips for one emotion (e.g., happiness) shows different movements. Even so, in all 10 clips of one emotion, that emotion was proven by Atkinson to be recognizable, providing an ideal data set for analyzing what specific movement components were being perceived as associated with each emotion. We developed our method to identify which movement components were essential to the expression and experience of these four basic emotions based on Atkinson’s clips.

Our first task was to identify the variables likely to be significant, and to reduce the number of variables down to a testable number from the numerous potential variables, in order to make possible the statistical analysis necessary to scientifically determine which components contribute to the elicitation of each emotion.

To achieve this task, we began with a 3-step protocol established (as early as 1970) by Martha Davis for analyzing patterns to identify important variables, which became our Stage A: (1) Look at all the material under consideration repeatedly to identify what motor components (variables) are important to study, and eliminate those which are minimal or absent; (2) develop clear criteria for narrowing down the number of components to be studied to those most characteristic to the researched movement, (3) analyze small units of variations (in our case: Atkinson’s short video clips, which included 10 different variations of motor expressions of each emotion, i.e., 10 different movements in 10 different clips), to learn how components within them appear to cluster (Davis, 1970). These three steps were carried out by Certified Movement Analysts (CMAs), experts trained in detailed observation and coding of LMA movement components, and in recognizing patterns of meaning in the phrasing and inter-relationship of those components.

Our method added three new steps to this protocol to integrate the best analysis from observation by CMAs with testing the effects of movement on participants:

Stage B: (4) Create Motifs (LMA symbols) of different combinations (clusters) of motor components extracted from motor expressions of the same emotion; (5) Conduct pilot studies to quantify participant’s emotional experience in response to moving the different Motifs with a forced-choice questionnaire, and based on the results, reduce the number of Motifs and components to be analyzed; (6) Code the prevalence of movement components within each Motif to enable quantifying their effects, for statistical analysis.

### Stage A: Using Martha Davis’s 3-Step Protocol

#### Step A1–2: Identification of Motor Components (Variables) and Criteria

**Figure 2:**
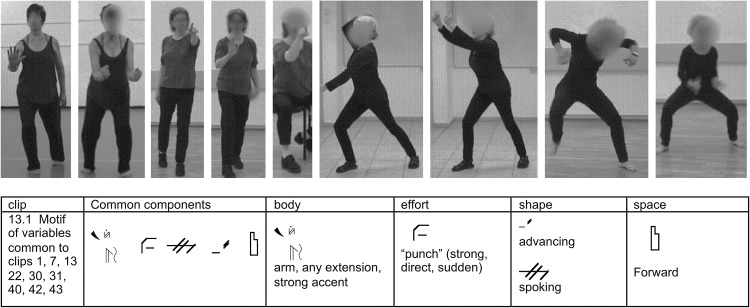
In the Common Components analysis, CMAs watched all 10 of Atkinson’s 3-s clips of the same emotion, looking to see if any components appeared in all or most (at least 8/10) of the clips. These components are depicted with Horizontal Motif, which can show the essential components of a movement in sequence. **(Top row)** Still shots of CMAs moving Motif components found in the main action of Atkinson’s clips 1, 7, 13, 22, 31, 40, 42, and 43. **(Bottom row)** A horizontal Motif of the common components found in the main action of 9 Atkinson clips of the same emotion, Anger. The common components motifed here are: Body–Arm extends; Effort–Punch (Strong, Sudden, Direct); Shape–Spoking and Advancing; Space–Forward; Phrasing–Strong Accent Emphasis.

We used two different methods to identify the prevalent movement variables and eliminate those appearing minimally or absent. In the first, the “Common Components” method, two experienced CMAs viewed all of Atkinson et al. (2004) clips, annotating components common to all 10 motor emotional expressions of the same emotion with a horizontal Motif (a form of Motif writing which identifies the essential and main components of a movement segment; Figure 2). They discussed their observations using clear criteria for inclusion of components as variables: repetition, frequency, duration, and emphasis, to reach consensus regarding which were the “common components” to study. Repetition means that a component appeared several times in a row in the movement, for example, 3 bounces in the last second of a Happiness clip. Frequency refers to how often components appear, such as one or both arm extending partially or fully forward in all the angry clips. Duration criteria let us know if the component was fleeting or prevailed in the movement, and Emphasis criteria were used in looking at the main action of the movements, rather than looking at the preparatory or follow-through stages of the clip.

**Figure 3:**
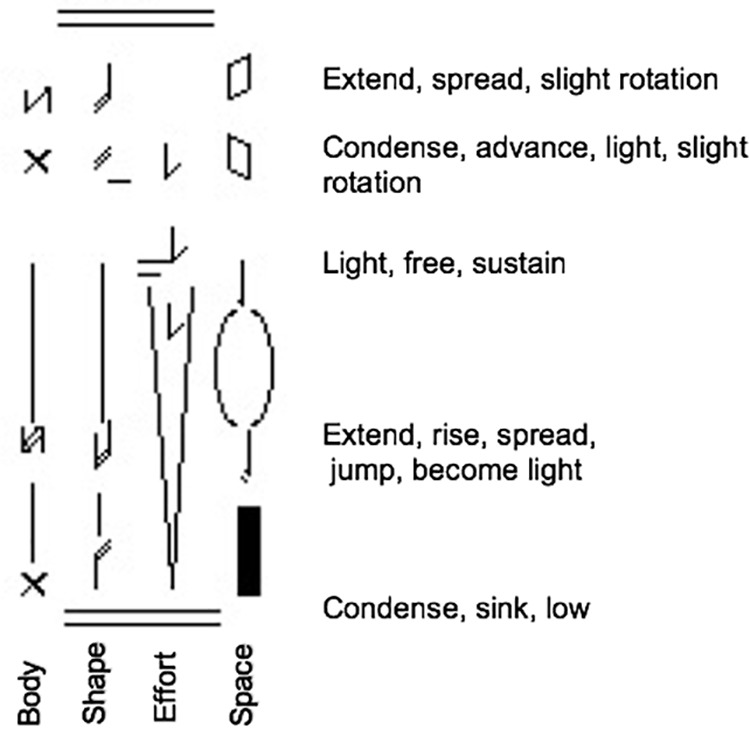
A vertical Motif of one of Atkinson’s clips with the highest recognition of Happiness. Vertical Motif was used for the micro-analysis method to capture details in a column each for symbols from Body, Effort, Space, and Shape, depicting the moment of onset in the phrase and duration of each prevalent component throughout the movement. This method facilitates analysis of the duration, phrasing, co-occurrence or other nuances in the relationships among components. By motifing each category in its own column, researchers can later test which category might be significant, e.g., by giving movers just one column to move.

In the second, “Micro-analysis” method for identifying the prevalent movement variables, two clips for each emotion (one performed by a male actor and one performed by a female actor) were motifed in detail by two experienced CMAs. The clips selected for this analysis were those having the highest recognition rate for that emotion in Atkinson’s study. The CMAs observed, and motifed these eight sample clips (2 for each of the 4 emotions), using LMA Vertical Motif to capture details, annotating for each clip its unique combinations of Body, Effort, Space, and Shape characteristics. In the Vertical Motif, there was a column each for symbols from Body, Effort, Space, and Shape, precisely annotating the presence and duration of each prevalent component throughout the movement, so the co-occurrence or other relationship of components during the movement could be readily seen (Figure 3). An advantage of Vertical Motif over Horizontal Motif is its ability to note nuances which might be important to the context of expression, such as each component’s duration, phrasing (i.e., how the components appear, shift, disappear in preparation to move, the peak of movement and follow through, etc.), in relationship to components in other columns, making obvious any timing overlaps in the appearance/disappearance of components that are not exactly concurrent. This micro-analysis of two ‘variations’ of motor expression (two clips) of each emotion was intended to find out how movement components appeared to cluster in the observed movement, and to pick up non-component variables such as sequence of the components, combinations of them or duration of them, etc.

#### Step A3: Movement Analysis for Clusters and Patterns of Components

Using both the above methods, we analyzed variations of samples to find how variables appeared to cluster: first, by comparing all samples of the same emotion, we isolated only the variables common to all samples. Second, by analyzing in detail two validated (by Atkinson et al., 2004) representative samples, we hoped to determine if phrasing, duration or relationship among variables might be significant.

### Stage B: Using Motif to Test Combinations of Variables Identified in Stage A

#### Step B4: Creating Motifs

**Figure 4:**
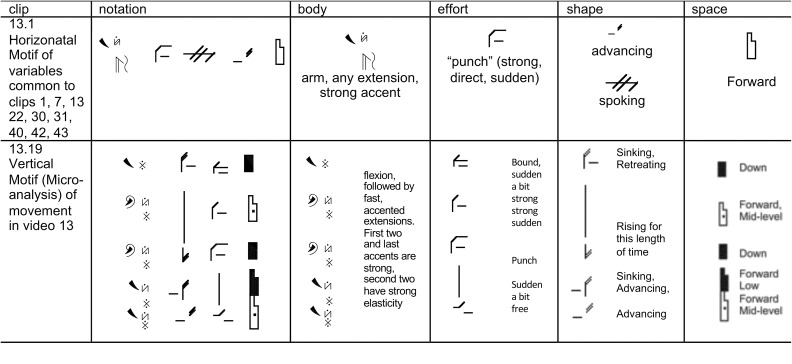
Depicts Motifs from Stage A3, Movement Analysis for clusters and patterns of components. In this stage, one can graphically see the common components, and also where and how they appear in a whole movement phrase.

**Figure 5:**
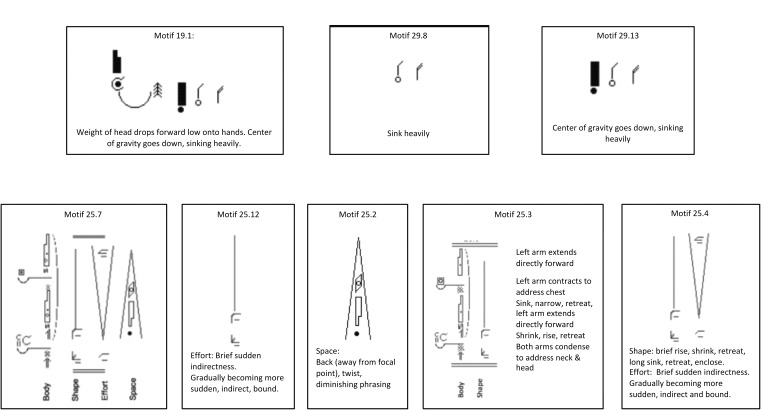
Examples of the combination-Motifs: the **(top row)** shows horizontal Motif of the common components method (19.1) for Sadness, and combinations of two or three components for testing variables (29.13 and 29.8). The **(bottom row)** shows a vertical Motif sample micro-analysis method of clip 25 (25.7) for Fear, and parts of it separated into columns for testing, such as single constituent components (the Effort symbols column only in 25.12 or the Space symbols only column in 25.2), or two columns testing: Body and Space Symbols together in 25.3, and Shape and Effort in 25.4.

We took all the prevalent components identified in the first stage that withstood the criteria of the “common component” method, and all the components of the vertical Motifs of the two best recognized clips of each emotion, and created combination-Motifs of single constituent components (for example, the Effort symbols only, Figure 4), and combinations of two or three component types (for example, in the vertical Motif, a column of the Effort and Shape symbols). In creating these combinations of components (some of which we hoped would elicit the associated emotion when moved) we took into account our findings from the analysis of the vertical Motifs: e.g., which components tend to appear together, which tend to appear one after another in a certain sequence phrasing, etc. We used Motif to organize the components into combinations for testing, in order to ask people to move them and to examine their emotional response to the movement (Figure 5). All together, the Motifs of combinations of components (both horizontal and vertical Motif) and the full vertical Motifs of the 8 best recognized clips of each emotion) resulted in 33 different Motifs for anger, 22 for fear, 33 for sadness, and 40 Motifs for happiness.

#### Step B5: Testing the Combinations in Pilot Studies to Further Reduce the Number of Variables

We tested the combination Motifs in two pilot studies. All participants of the pilot studies, and the later online study, joined the study voluntarily, and signed a written informed consent form prior to taking part in the study. This study was carried out in accordance with the recommendations of University of Michigan HRPP (Human Research Protection Program) Operations Manual. The protocol was approved by the Institutional Review Boards of the University of Michigan Medical campus. All subjects gave written informed consent in accordance with the Declaration of Helsinki.

The first pilot study was conducted with 10 expert LMA readers, during one session of 2.5 h, and another session of 50 min. Although our pilot study pool was small, the participants represented a multi-cultural sample of people: 6 North Americans, 2 from South or Central American, 1 from Eastern Europe, and 1 from Western Europe.

Participants in the first pilot read the Motifs generated in step 4 above, one after another. They were asked to read and move the components depicted by each Motif in their own way until the movement elicited an emotion. These readers’ expertise was sufficient to accurately read and move from the Motifs, after which they answered a forced-choice questionnaire in which they indicated which, of the emotions Happiness, Sadness, Fear, Anger, Neutral/no emotion, they felt when moving the Motif, and what intensity their emotions were following the movement, on a scale of 1 (very weak) to 5 (very strong.). In the first pilot, participants received an envelope with 4 Motif variations from the same emotion. They moved each Motif, answered the questionnaire about which emotion (if any) they felt moving it and its intensity, then received a new envelope containing another set of four Motif variations of another emotion, until all the envelopes were read by the participants.

By moving directly from symbols, as opposed to watching a video or learning to move the combination of movement components from another person, the participants’ impression from each motor component was ‘uncontaminated’ from any unintended influence of co-occurring other motor components as might occur through live or recorded demonstration.

**Figure 6:**
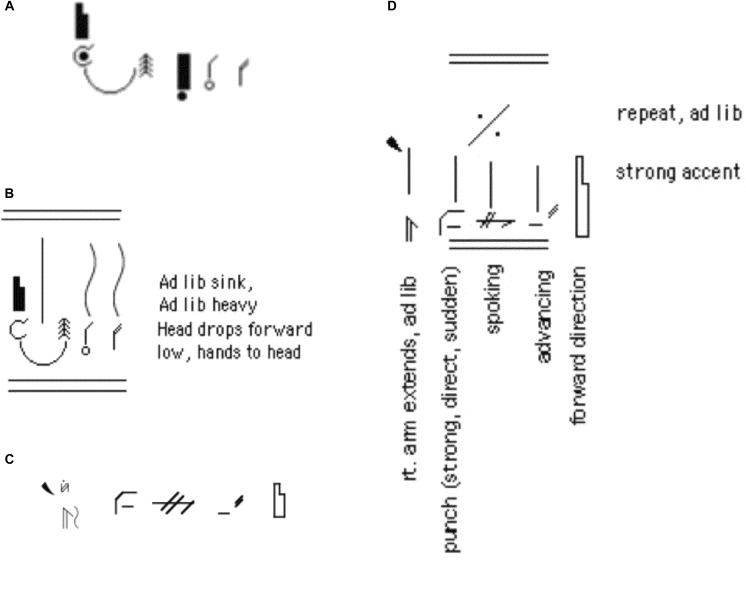
Examples of converting horizontal Motifs to vertical to show duration. **(A)** Shows the common components of Sadness in horizontal Motif, which doesn’t show duration. **(B)** Shows these common components of Sadness converted to vertical Motif for testing, with strokes showing duration. **(C)** Shows the common components of Anger in horizontal Motif, which doesn’t show duration, converted to vertical Motif in **(D)**, to depict duration and repetition of the action. Most of the Anger video clips showed short, repeated actions, rather than one long one, leading us to motif repetition rather than only a long duration in this conversion.

Results from the first pilot indicated that recognition of Happiness from horizontal Motifs (common components method) was less robust than recognition from vertical Motifs, indicating that the set of symbols in the common components had not yet been clearly identified. Thus, three additional CMAs were asked to each view all 10 clips for each emotion and Motif components predominating the clips of that emotion. Their Motifs were compared to those generated earlier, and any components they identified as possibly significant, which were not included earlier, were added. Following this identification of additional predominating components, we added 2 new full Motifs (of common components) from this second CMA team’s analysis, and Motifs of combinations of their components, to the set we were testing in Pilot 2. Also, results from the first pilot indicated that duration of the movement was significant, but reading single Motif symbols in horizontal Motif seemed to be confused with reading symbols of short duration, as in vertical Motif. In the cases where the horizontal Motif didn’t score high on enhancement of the correct emotion (such as for common components of happiness), we wondered if motifing the duration might matter. Therefore, for the next round of testing, we converted the Motif of the common components from horizontal Motif to vertical, extending the length of each symbol (to show its duration) with an ad-lib action stroke (a Motif symbol for “continue to move in this way at liberty”) for a longer duration, to match the length of similar vertical Motif. Sometimes we inserted a repeat sign, signaling readers to do the movement more than once (Figure 6). For the second pilot, we also eliminated Motifs that the first pilot clearly showed unsuited for further study, i.e., Motifs that hardly evoked any emotion. This procedure resulted in having 13 Motifs for each emotion. Lastly, in this second pilot, participants received Motifs for all 4 emotions in each envelope, thereby removing the chance that they might infer the emotion of one Motif from moving the previous one.

In the second pilot, eight additional readers were asked to read and move the new set of Motifs. The readers’ expertise was sufficient to accurately read and move from the Motifs, after which they answered the same forced-choice questionnaire as in the first pilot.

Descriptive results from the two pilot studies indicated that several Motifs did not evoke the original emotion (i.e., the emotion expressed in the clips from which the components in the Motif were taken) by any participants. These Motifs were eliminated and reduced the overall number of Motifs that were used in the final study to 40:

9 Motifs composed of components originating in movements expressing anger,

8 Motifs composed of components originating in movements expressing fear,

13 Motifs composed of components originating in movements expressing happiness, and 10 Motifs composed of components originating in movements expressing sadness,

However, the number of components in those Motifs was still too large for statistical analysis and additional steps were taken to reduce it to 32 for coding: decisions were made to code some similar components under one variable. For example, some Motifs depicted specific areas of the torso as the Body component “Chest,” while others depicted larger areas of the torso with the symbol for “Core.” We decided to unite them under one variable, so that all Motifs for “Chest” were coded simply as “Core,” and when either the chest or core component appeared in a Motif, it was considered as this “core” variable. The Motifs for chest expanding and core to distal expanding were also combined into one variable of expanding in general. Similarly, the Motif for Center of Gravity dropping down and the component of sinking were combined into one “sinking” variable for coding, so either or both would be recorded as one variable, and the component of Rotation in Space and twisting the Body were combined into one coded variable of Rotation. Lastly, in the happy Motifs, Buoyancy and Re-initiating Phrasing/rhythmic repetition were condensed into one variable: Rhythmicity.

Of the 32 variables coding the LMA components in the remaining 40 Motifs that were moved by the study participants, two did not appear at all, and one appeared only once. Thus, they were not included in the final analysis, which was therefore based on only 29 variables.

#### Step B6: Quantification of the Data Onto the Coding Sheet

In our study, participants rated their emotion in response to moving each Motif. These Motifs were each composed of a different combination of several components, where each component appeared in several different Motifs. We were interested in knowing the effect of each LMA component on the participant’s emotional state. Thus, our variables for the statistical analysis were the components and not the Motifs, even though the emotional scores were related to Motifs. We therefore had to establish a way to “transform” the quantitative emotional ratings we got for each Motif into quantitative rating that related to each LMA component. We did that based on the “coding sheet” that we created, in which we “quantified” the amount of each component within each Motif, so that for the purpose of the statistical analysis, the emotional rating of each participant for any specific movement component (variable) was calculated as the product of multiplying the emotional rating for each Motif by the “amount” (prevalence) of that component within that Motif.

We organized our coding sheet so that we could code components for each Motif within the categories of Body, Effort, Space, Shape, and Phrasing in columns, which concurred with the columns of Motif combinations that were tested.

Nine Effort variables—Flow Effort: Bound and Free; Time Effort: Sudden and Sustained; Space-Effort: Direct and Indirect; and Weight Effort: Strong and Light, plus One Effort-related Body variable: the lack of weight activation: Passive Weight/Heavy.

Eight Shape variables—Expand, Condense; Rise, Sink; Spread, Enclose; Advance, Retreat.

Seven Space variables —Up, Down; Forward, backward; Side Open, Side Across, and

Rotation (which could be a Space component or a Body action), and was combined with twist as one variable.

Five Body components were coded—three Body-parts: core, arms and head; and two Body-actions: arm(s) to upper body, and jump.

Three Phrasing components—Increasing; Decreasing and Rhythmicity (a combined variable of Reinitiating, repeated movement and buoyant accents).

Of the 32 variables on the coding sheet, the 3 that were eliminated before statistical analysis were: Side open and Side across (which were not present in any of the Motifs that stayed in the final study) and Decreasing intensity phrasing (which appeared in only one of the Motifs that were tested in the final study) (see Coding Sheet in Supplementary Material).

The original Atkinson’s emotional expression video clips, which served as the origin for the vertical Motifs, lasted 3 s. Thus, we divided each vertical Motif (in which motor components are annotated over time) into three equal segments, each representing one second of the movement, and checked to what extent each component appeared in each segment. Components that appeared in a segment of the Motif were coded as 1 for that segment, and those that didn’t appear were coded as 0. Because horizontal Motifs do not annotate timing, to establish comparable units for the vertical and horizontal Motifs, short horizontal Motifs were also divided into three segments and were coded as if each component in them lasted for all three seconds, to match the coding of the vertical Motif: This was appropriate because participants moved each Motif as long as needed to perceive its associated emotion.

In this way, every Motif had a total score for each component quantifying its prevalence in the Motif. This score had a value of 0 if the motor component didn’t appear in the Motif at all, a value of 1 if it appeared in up to one third of the duration of the Motif, the value of 2 if it appeared during two thirds of duration of the movement annotated in the Motif, and the value of 3 if it appeared along the entire duration of the movement. Thus, every Motif was coded from 0 to 3 for all 29 components.

Once we had our final decision regarding which Motifs and components to use, the Motifs, along with words describing each component in them, were entered into a Qualtrics questionnaire, that was distributed through the internet, so that any potential participant, anywhere in the world, who met the study criteria could take part in the research, read the Motifs, move them and rate his/her emotional response. Language instructions for specific aspects of qualitative movement (such as “lighten up”) have been demonstrated as effective at producing measurable changes in the movement of 20 study participants (Cohen et al., 2015). Thus, in the Qualtrics questionnaire, we added to each Motif a short language description of each component included in it, so participants could read the components in Motif or language, according to their skills. Overall, 80 people participated in the study. Their inclusion/exclusion criteria and demographic details can be found in Shafir et al. (2016).

### Data Analysis

To determine which motor components contributed to the enhancement of each emotion, a logistic regression model was fitted to predict each emotion (anger, fear, happiness, and sadness) felt during the movement of a certain Motif, using each motor component (variable) score as a predictor. Because each participant performed multiple Motifs, we used a GEE (Generalized Estimating Equations) model to take into account correlations among the responses for each participant. To adjust for the multiple tests for each emotion, we applied the Bonferroni correction to the *p*-values, so that *p*-value of 0.0017 (0.05/29 = 0.0017) or less was considered to be statistically significant. The results of this analysis were published in Shafir et al. (2016).

## Limitations

The most significant limitation of our method is that it is limited to the components that were predominant in the original samples used. In our study, all of the components we tested were taken from Atkinson’s (2004) clips, i.e., from motor expressions of only 10 different people. These, however, might not have included all possible motor components potentially associated with each emotion and may not be representative for all people. Thus, there might be additional motor components which we never tested that are also capable of enhancing emotions. In addition, some decisions for data reduction, based on the data we had, might have been wrong: for example, today in hindsight, after testing our results in a new study (Melzer et al., 2018) evidence that combining all rotation types as one variable meant that rotation did not turn up as a statistical predictor for the emotions tested. Retesting this variable in Melzer et al. (2018) found that Rotation (revolving in space) may be associated with Happiness, while twist may be better associated with Fear. Our method to reduce the number of variables through pilot studies, where expert LMA readers moved various combinations of motor components, may have another significant limitation: Any testing done by participants moving relies upon their ability to move/improvise the distinct components they read either from Motif or language descriptions of the movement components. Research hasn’t been done on different ways to use Motif in quantitative research, so there are some unknowns: how can a study take into account differences in skill level or cultural differences of those reading and moving the Motifs? In reading multiple Motifs, how does the previous reading influence successive readings? How well do movement outcomes for people who read Motif or language descriptions of LMA components represent social and cultural diversity? Most of the LMA expert readers who participated in our pilot studies were from North America. These questions indicate that the specific people who participated in the pilot studies might have affected which variables we chose to keep during the process of reducing the number of variables, and thus it might have affected the final results as well, even though the population participated in the final study was diverse. Lastly, as is the case for many research methods, an additional limitation of this method is that it requires a researcher with sufficient expertise in the method, here Laban Movement Analysis, to be involved in the study to carry out a similar sophisticated and extensive methodological process or adapt it to the needs of other studies.

## Conclusion

While the research record of what LMA can track and quantify in individual expression or prescribed movements performed by multiple movers has been demonstrated by numerous researchers (see literature review in the section “Introduction”), the nature of expressive unscripted movement has been difficult to investigate quantitatively due to the enormous number of variables involved. The multi-stepped approach described here can bridge a gap in methods for observing improvised (unscripted) movements to identify recurring patterns in human movement behavior among multiple movers, and as such, can be valuable for research in the social sciences.

The main novelties in the methods we used to quantify the movement components compared to previous studies that used LMA are: first, developing three new steps (described in stage B) beyond those suggested by Davis (1970) used in Stage A. These new steps are: Step (4) Creating Motifs of combinations of elements, which enabled us to study unlimited numbers of movements containing specific motor components instead of using limited number of specific motor sequences, as well as using clean data, uncontaminated by co-occurring movement components that might be unintentionally introduced through live or video demonstration; Step (5) Testing the combinations in pilot studies and using their results to reduce the number of variables; and Step (6) Quantification of the data based on the coding sheet, and the use of logistic regression for data analysis. Second: using internet-based tools (Qualtrics software and email LISTSERV) to collect survey data from all over the world, which enabled to increase both the number of expert participants and the participants’ cultural diversity.

The methods described in this paper suggest that expert observers (such as CMAs), who are trained to see the nuanced, qualitative components of expressive movement, are crucial to teasing out, coding and quantifying testable variables for statistical analysis. We hope this method can be used by others to test how the effects of unfolding movement changes contribute to their therapeutic, communicative, or expressive intent, and to quantify them for research, not only in DMT, but in any field to which movement is of interest. With this method, dance/movement therapists, behavioral scientists and researchers in the arts can do justice to their sophisticated expert observations of movement’s content in context.

## Author Contributions

RT was the Certified Movement Analyst for this study, primarily responsible for its movement design, developing the movement aspects of the methodology stages, recruiting volunteers for the pilot studies, overseeing CMA consultants, carrying out the movement analysis protocols discussed here, and interpreting the various stages of movement data. TS conceived of the original study and was its PI. She designed and directed the overall study, science processes and analysis, and all statistical analysis, co-designed all phases of the study, and was first author of the paper describing the entire study. RT wrote the manuscript. TS contributed to writing and revising the manuscript. RT and TS approved the manuscript and agreed to be accountable for all aspects of the work.

## Conflict of Interest Statement

The authors declare that the research was conducted in the absence of any commercial or financial relationships that could be construed as a potential conflict of interest.

## Supplementary Material

TABLE S1 *Quantifying unscripted movement with LMA* Coding Sheet.
